# Does carer psychological inflexibility moderate the relationship between motor neurone disease symptomatology and carer anticipatory grief emotions?

**DOI:** 10.1177/13591053251328490

**Published:** 2025-03-29

**Authors:** Ana Paula Trucco, Mizanur Khondoker, Naoko Kishita, Tamara Backhouse, Thomas M Meuser, Eneida Mioshi

**Affiliations:** 1 University of East Anglia, UK; 2 University of New England, USA

**Keywords:** behavioural changes, carers, disease severity, motor neurone disease, psychological inflexibility

## Abstract

Anticipatory grief (AG) in family carers of people living with motor neurone disease (MND) is underexplored. Research has identified MND symptoms as significant predictors of AG in carers. This study investigated whether carer psychological inflexibility moderates the relationship between MND symptoms and carer AG, a crucial area for informing supportive interventions. Two moderation analyses with 75 carers (UK = 70, USA = 5) were conducted. The first analysis found that while MND disease severity (ALSFRS-R) and psychological inflexibility (AAQ-II) were associated with AG (MMCGI-SF), psychological inflexibility did not moderate this relationship. Similarly, the second analysis revealed that while behavioural changes (MiND-B) and psychological inflexibility influenced AG, the interaction between them was not significant. These findings suggest that although psychological inflexibility does not moderate the relationship between MND symptoms and carer AG, it may still impact carers’ emotional distress, highlighting the need to address this in interventions. Clinical implications are discussed.

## Introduction

Motor Neurone Disease (MND) is a neurodegenerative and progressive disease, with heterogenous onset and progression. It primarily affects the upper and lower motor neurones, leading to advancing motor and extra-motor symptoms (Hardiman et al., 2017). Traditional MND phenotypes can be classified based on the extent of upper and lower motor neurone involvement, with amyotrophic lateral sclerosis being the most common phenotype (Statland et al., 2015). Common motor symptoms of MND are associated with motor dysfunction, such as muscle weakness, muscle wasting, dysphagia, cramps and respiratory insufficiency (Hardiman et al., 2017). Additionally, cognitive and/or behavioural symptoms are observed in over 50% of people living with MND, with approximately 15% experiencing concurrent frontotemporal dementia (Strong et al., 2017). Cognitive impairment has been typified by executive dysfunction, deficits in social cognition and verbal fluency, language dysfunction and memory deficits (Abrahams et al., 2000), with verbal fluency emerging as the most consistently observed cognitive change (Abrahams, 2023). Behavioural symptoms include apathy, disinhibition, stereotyped behaviour, rigidity and emotional lability (Trucco et al., 2024a). Life expectancy for people living with MND ranges from 2 to 3 years from symptom onset, there is no cure to date and present treatments are limited (Kiernan et al., 2011).

Research has revealed that providing care for someone living with MND can significantly impact the emotional wellbeing of the primary carer, usually a close relative or family member (Pinto et al., 2021; Trucco et al., 2024c). Carers face a series of changes and losses throughout the trajectory of this disease, often experiencing anticipatory grief (AG). AG is a normal grief response motivated by the perception of past and present losses and changes, and expectations of future losses culminating in death (Rando, 1986), and a complex experience that carers face involving the relationship with the care recipient, the changes resulting from the losses and the carers coping with this new reality (Nielsen et al., 2016). It has been suggested that various illness trajectories may influence carers’ AG experience differently (Coelho and Barbosa, 2017). Due to the rapid progression and uncertainty that accompanies MND, it is important to delve into the experience of AG in carers of people living with MND, as their support needs are likely distinct from other populations.

A previous systematic review (Trucco et al., 2023) revealed that factors associated with AG in carers of people living with MND include the uncertainty of the disease, carer anxiety and depressive symptoms, changes in carer-care recipient relationship and a diminished social life. This review highlighted that the majority of existing studies in this area appear more focused on post-death grief and prolonged grief disorder (PGD) than present AG symptoms. A recent study indicated that MND disease-related factors (i.e. disease severity and behavioural changes) are more significant predictors of AG emotions, rather than carer-related factors (Trucco et al., 2024b). This presents a challenge for this carer population, as many disease-related factors are inherently non-modifiable at present.

It is crucial to better understand how MND carers regulate and process their feelings and emotions; as grief during caregiving has been found to be a risk factor for poor bereavement outcomes (Nielsen et al., 2016) and when not processed adaptively, there is high risk of developing PGD (Aoun et al., 2015). AG is real grief, often no different in intensity or quality from post-death grief (Meuser and Marwit, 2001). Importantly, the prevalence of AG among family carers of persons living with a life-threatening illness, such as MND, was found to be higher than post-death grief in the general adult population (Kustanti et al., 2022). Consequently, investigating potential modifiable factors that could moderate the relationship between MND symptomatology and carer AG grief emotions seems essential for providing better support to carers and informing future clinical non-pharmacological interventions.

Psychological inflexibility may play a critical role in explaining the impact of MND symptomatology on carer AG reactions. Psychological inflexibility refers to the attempt to decrease internal private experiences, such as thoughts and feelings, even when doing so is inconsistent with personal values (Hayes et al., 2006) and can significantly influence coping strategies. When MND carers are faced with symptoms of MND, these can often act as stressors and could lead to increased levels of negative thoughts and feelings. When a carer is presenting with higher levels of psychological inflexibility, they tend to devote a lot of effort and energy to controlling and suppressing such negative thoughts and feelings (e.g. denial) rather than accepting and embracing them. This avoidance strategy – one of the processes of psychological inflexibility (Hayes et al., 2013) – could contribute to worse mental health outcomes (e.g. anxiety, stress). It has been associated with negative outcomes such as greater disfunction and increased distress including prolonged grief disorder – a persistent grief response accompanied by intense emotional responses (e.g. sadness, anger, denial), where disturbances result in significant distress or impairment in important areas of functioning (Baker et al., 2016). Research has suggested that when grief emotions are avoided, these cannot be processed, and as a consequence they remain high (Coelho et al., 2018). While avoidance may offer temporary relief, it is essential to address it, as it does not provide sustainable coping mechanisms (Karekla and Panayiotou, 2011; Ottenbreit and Dobson, 2004). In addition, a previous study reported that MND carers who demonstrate greater acceptance and adaptability – core aspects of psychological flexibility, the conceptual opposite of psychological inflexibility – to their reality are able to cope better with challenges MND may bring to affected families (Trucco et al., 2024c).

A recent systematic review (Han et al., 2021) explored the effectiveness of Acceptance and Commitment Therapy (ACT), an intervention targeting psychological inflexibility on mental health outcomes in family carers. The review found that ACT effectively reduces anxiety, depressive symptoms and stress among the carer population. However, studies addressing AG remain limited. For example, a recent systematic review (Jones et al., 2022) that explored the effectiveness of ACT for managing grief, identified only two eligible studies, both focused on bereaved family carers of patients in palliative care, rather than AG specifically. Thus, no studies have directly examined the impact of psychological inflexibility in AG among MND carers, highlighting a significant gap in the available scientific literature. While AG and psychological inflexibility have been studied independently, their interaction in the context of MND carers remains relatively unexplored.

Consequently, to address this research gap, this cross-sectional study examines psychological inflexibility as a moderator variable capable of explaining the relationship between MND symptomatology and carer AG emotions. This study builds upon a previous research study (Trucco et al., 2024b), which involved the same cohort and served as a foundational analysis. While both studies draw on data from three out of four instruments (Marwit-Meuser Caregiver Grief Inventory-Short Form, ALS Functional Rating Scale-Revised, Motor Neurone Disease Behavioural Instrument), the present study addresses a distinct research question and extends the analysis by specifically exploring the role of psychological inflexibility to provide novel insights that could inform targeted interventions.

## Materials and methods

### Participants and procedure

This cross-sectional study recruited 79 family carers currently supporting people living with MND who were 18 years or older and provided unpaid care. Recruitment was conducted from July 2021 to February 2023, commencing in the United Kingdom (UK) and subsequently expanding to include United States of America (USA) in October 2022 due to challenges encountered in recruitment. The process involved disseminating study information through MND/ALS Associations, carers’ support groups and various social media platforms such as Twitter (@FactorMND). In the UK, two tertiary hospitals, namely Norfolk and Norwich University Hospitals NHS Foundation Trust and Sheffield Teaching Hospitals NHS Foundation Trust, also facilitated recruitment by distributing the study information via leaflets, participant information sheets and social media posts.

Participants had the option to complete a survey either through an online platform or in paper format. Online survey data were collected and managed by the Joint Information Systems Committee electronic data system, ensuring anonymity for all participants.

Ethics approval for the present study was granted by the West Midlands – Black Country Research Ethics Committee (UK) for UK participants [IRAS 281943; REC 20/WM/0185] and by the Faculty of Medicine and Health Sciences Research Ethics Subcommittee from the University of East Anglia (UK) for USA participants [ETH2223-0204].

### Instruments

#### Carer anticipatory grief

The Marwit-Meuser Caregiver Grief Inventory-Short Form (MMCGI-SF; Marwit and Meuser, 2005) was employed to measure the grief experience in carers. Each of the 18 items is rated on a 5-point Likert scale ranging from 1 = strongly disagree to 5 = strongly agree, with a score range from 18 to 90. Scores in the average range represent common responses to loss in the carer experience, high scores (i.e. 1+ SD above the sample mean) may indicate the need for support and low scores (i.e. 1+ SD below the sample mean) may mean positive coping adaptation or denial. The Cronbach’s alpha for the current study was 0.94.

It is important to note that for the purpose of this study, one item from the scale was modified with the author’s permission (TM) to better suit MND carers, as the original wording was tailored for dementia carers.

#### Carer psychological inflexibility

The Acceptance and Action Questionnaire-II (AAQ-II; Bond et al., 2011) was used to assess the degree of carer psychological inflexibility. Each of the seven items is rated on a 7-point Likert scale ranging from 1 = never true to 7 = always true. The items of the AAQ-II include the following statements ‘My painful experiences and memories make it difficult for me to live a life that I would value’, ‘I’m afraid of my feelings’, ‘I worry about not being able to control my worries and feelings’, ‘My painful memories prevent me from having a fulfilling life’, ‘Emotions cause problems in my life’, ‘It seems like most people are handling their lives better than I am’ and ‘Worries get in the way of my success’. With a normative mean of approximately 25 or above, the maximum score is 49, where higher sum scores indicate greater psychological inflexibility. The Cronbach’s alpha for the current study was 0.93.

#### MND disease severity in the person living with MND

Disease severity was assessed by the revised version of the ALS Functional Rating Scale (ALSFRS-R; Cedarbaum et al., 1999). This measure comprises four domains including bulbar function, fine motor function, gross motor function and respiratory function and reflects motor impairment and disability progression. Each of the 12 items is rated on a 4-point Likert scale ranging from 0 = no function to 4 = normal. Maximum score is 48; lower scores denote greater disability. The Cronbach’s alpha for the current study was 0.86.

Carers were responsible for evaluating the ALSFRS-R. While this measure has initially been developed to be scored by healthcare professionals, past studies (Mehdipour et al., 2023; Meyer et al., 2023; Miano et al., 2004; Montes et al., 2006) have shown high reliability in ALSFRS-R assessments conducted by healthcare professionals, carers and people living with MND themselves. Moreover, the ALSFRS-R utilised in our research offered explicit scoring instructions to be comprehensible to lay-individuals, thus ensuring accessibility.

#### Behavioural changes in the person living with MND

Changes in behaviour were measured by the Motor Neurone Disease Behavioural Instrument (MiND-B; Mioshi et al., 2014), completed by the carer. The instrument comprises three subscales: disinhibition, apathy and stereotypical behaviour. Each of the nine items is rated on a 4-point Likert scale ranging from 1 = everyday to 4 = no changes from normal behaviour. The cut-off score indicating presence of behavioural changes is <34; with lower scores representing greater behavioural symptoms. The Cronbach’s alpha for the current study was 0.87.

#### Demographic information

In conjunction with the utilisation of standardised assessment tools, demographic data pertaining to both the carer and the person living with MND were systematically collected. For carers, information included age, gender, length of time providing care, country of residence and relationship to the care recipient. Regarding the person living with MND, data collection provided by the carer encompassed age, MND phenotype classified based on the distribution and severity of upper and lower motor neurones, including ALS, progressive bulbar palsy, progressive muscular atrophy, primary lateral sclerosis (Statland et al., 2015). Additionally, according to the classification proposed by Strong et al. (2017), we included MND-frontotemporal dementia (MND-FTD) and months since their initial diagnosis.

### Statistical analysis

Of the 79 participants recruited (UK = 74; USA = 5), four participants partially completed the measures for this study. This resulted in a dataset of 75 family carers (UK = 70; USA = 5).

Descriptive analyses of demographic information were performed to characterise the sample of carers and people living with MND. Pearson’s *r* correlations were conducted to assess relationships between potential covariates (carer age and length of care) and the independent (ALSFRS-R; MiND-B), moderator (AAQ-II) and dependent (MMCGI-SF) variables. These correlations also served as a preliminary check for multicollinearity, with a threshold correlation coefficient of >0.70 among two or more variables indicating potential multicollinearity between variables (Shrestha, 2020). Control, independent and moderator variables that demonstrated significant correlations with AG at a *p* level of <0.05 were included in the moderation analyses. To further evaluate multicollinearity, Variance Inflation Factor (VIF) values were calculated for all predictors in each moderation model, including the interaction term. Participant’s country of residence was included as a covariate in the models to account for any potential differences between responses between the two countries, ensuring that the moderation effect was not confounded by country of residence.

**Figure 1.**
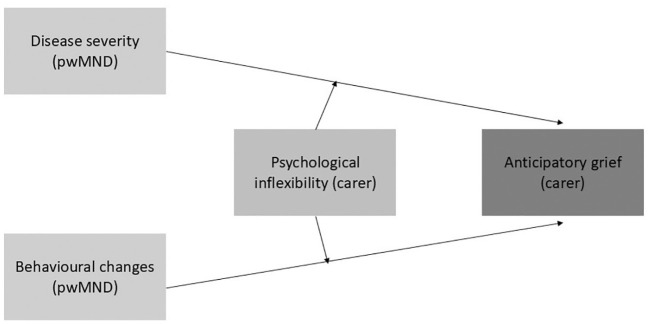
Conceptual framework. pwMND: people living with MND.

To investigate the moderating role of psychological inflexibility on the association between MND symptomatology and carer AG, two separate moderation analyses were conducted with severity of the disease and behavioural changes as independent variables, psychological inflexibility as moderator variable, carer AG as outcome variable and carer age and country of residence as covariates (Figure 1). The moderation effect of psychological inflexibility was assessed via statistical significance of the two interaction effects: ‘psychological inflexibility × severity of the disease’ and ‘psychological inflexibility × behavioural changes’ respectively. The PROCESS computation macro (Model 1; Hayes, 2013) was used to perform the analyses. All continuous variables included in the analyses were mean centred.

The analyses were performed using SPSS statistical software (Version 28).

## Results

### Participants

Descriptive statistics are reported in Table 1. The majority of participants were female carers (65.3%), spouses of the person living with MND (89.3%) and lived with the carer recipient (94.7%).

**Table 1.** Demographic characteristics and clinical variables of family carers and people living with MND (*n* = 75).

Family carer	*M* (SD), % or frequency
Age (mean, SD)	63.09 (10.46)
Gender (female %)	65.3
Relationship to pwMND (%)	
Spouse/partner	89.3
Parent	4
Child	5.4
Other	1.3
Living with pwMND (yes %)	94.7
Months caring (mean, SD)*	38.64 (44.08)
Hours providing care per week (%)	
<1–8	22.7
9–30	25.3
31–49	13.3
50–99	13.3
100 or more	25.4
Country of residence (frequency)	
United Kingdom	70
United States	5
Anticipatory grief (MMCGI-SF, %)	
High grief profile	22.6
Average grief profile	50.7
Low grief profile	26.7
Psychological inflexibility (AAQ-II)	18.99 (8.20)
People living with MND	Percentage or *M* (SD)
Age (mean, SD)	64.70 (11.74)
Gender (male %)	60
Phenotype of MND (%)*	
ALS	49.3
Progressive bulbar palsy	12
Progressive muscular atrophy	9.3
Primary lateral sclerosis	10.7
ALS-FTD	2.7
Don’t know	14.7
Months since diagnosis (mean, SD)	45.08 (47.88)
Disease severity (ALSFRS-R)	24.99 (9.11)
Behavioural changes (MiND-B)	30.13 (5.86)
Presence of behavioural symptoms	65.3%

* Missing data for months caring (*n* = 73/75), phenotype of MND (*n* = 74/75) and months since diagnosis (*n* = 73/75). MMCGI-SF (max 90) higher scores may indicate the need for support and lower scores may mean positive coping adaptation or denial; ALSFRS-R (max 48) lower scores denote greater disability; MiND-B (max 36) lower scores represent greater behavioural symptoms; AAQ-II (max 49) higher scores indicate higher level of psychological inflexibility.

The results derived from the MMCGI-SF revealed that approximately half of carers (50.7%) fell within the average grief profile, indicating they were experiencing common grieving emotions. In addition, 22.6% carers presented heightened and intense grieving emotions. Conversely, the remaining 26.7% were categorised within the low grief profile, indicative of adaptive coping mechanisms or potential denial of emotions. Total AAQ-II group mean score was 18.99/49 (SD 8.20), indicative of average and healthy levels of psychological flexibility (Table 1).

The majority of people living with MND in the study were male (60%) and, on average, the formal diagnosis of MND was conveyed within the previous 45 months. Behavioural symptoms were identified in nearly two thirds of the people living with MND (65.3%) and the mean for severity of the disease was 24.99 (SD 9.11; Table 1).

### Correlations and multicollinearity

Pearson’s correlations among demographic variables showed that carer age was negatively associated with AG emotions, which meant that being younger was associated with higher levels of grief. Therefore, carer age was controlled in the moderation analyses, in addition to country of residence.

No correlation between the control (carer anticipatory grief), independent (disease severity and behavioural changes) and moderator (psychological inflexibility) variables exceeded the recommended threshold of 0.70, resulting in no identified multicollinearity issues (Table 2). VIF values for each of the models ranged from 1.02 to 1.05 for the model with disease severity as the dependent variable, and from 1.03 to 1.19 for the model with behavioural changes as the dependent variable, further confirming the absence of multicollinearity.

**Table 2.** Pearson’s *r* correlations among variables (*n* = 75).

Variables	1	2	3	4	5	6
1. Carer age	1.00					
2. Length of care (months)	0.07	1.00				
3. PwMND disease severity	0.15	−0.08	1.00			
4. PwMND behavioural changes	0.35**	0.17	0.36**	1.00		
5. Carer psychological inflexibility	−0.31**	0.06	−0.19	−0.37**	1.00	
6. Carer anticipatory grief	−0.25*	0.05	−0.48**	−0.48**	0.50**	1.00

* Correlation is significant at the 0.05 level (2-tailed). **Correlation is significant at the 0.01 level (2-tailed).

#### Does carer psychological inflexibility moderate the relationship between MND symptoms and carer AG?

In two separate interaction analyses, we investigated whether psychological inflexibility was a significant moderator variable between disease severity and behavioural changes and carer AG. In other words, might high or low inflexibility differentially influence (moderate) carer grief responses?

##### Disease severity

Results showed that the main effect of severity of the disease on carer AG emotions was significant (*b* = −0.70, *p* < 0.01, 95% CI −1.01 to −0.38) and the main effect of psychological inflexibility on carer AG was also significant (*b* = 0.74, *p* < 0.01, 95% CI 0.38–1.10). However, the interaction effect between severity of the disease and AG was found to be non-significant (*b* = 0.03, *p* = 0.17, 95% CI −0.14 to 0.08), meaning no moderation effect of psychological inflexibility. The full model with severity of the disease and psychological inflexibility as independent variables explained 44% of the variance of carer AG (Table 3).

**Table 3.** Results of moderation analysis – disease severity.

Model	*b*	SE	*t*	LLCI	ULCI	*p*
Intercept	64.49	11.83	5.45	40.90	88.08	<0.001
ALSFRS-R_centered (X)	−0.70	0.16	−4.42	−1.01	−0.38	<0.001
AAQ-II_centered (W)	0.74	0.18	4.08	0.38	1.10	<0.001
ALSFRS-II × AAQ-II (X × W)	0.03	0.02	1.37	−0.14	0.08	0.174
Age (C1)	−0.06	0.15	−0.38	−0.35	0.24	0.701
Country of residence (C2)	−4.75	5.69	−0.84	−16.10	6.59	0.406
*R*^2^ = 0.44						
MSE = 144.024						
*F*(69, 5) = 10.66						
*p* < 0.001						

ALSFRS-R: assessment of person living with MND disease severity; AAQ-II: assessment of carer psychological inflexibility; LLCI: lower level of 95% confident interval; ULCI: upper level of 95% confidence interval.

##### Behavioural changes

Results showed that the main effect of behavioural changes (*b* = −0.93, *p* < 0.01, 95% CI −1.51 to −0.36) and psychological inflexibility (*b* = 0.66, *p* < 0.01, 95% CI 0.26–1.07) on carer AG were both significant. The interaction effect for this model was also non-significant (*b* = −0.00, *p* = 0.94, 95% CI −0.08 to 0.07). The final model, including behavioural changes and psychological inflexibility as predictors, and carer AG as dependent variable, explained 37% of the variance of carer AG (Table 4).

**Table 4.** Results of moderation analysis – behavioural changes.

Model	*b*	SE	*t*	LLCI	ULCI	*p*
Intercept	66.85	12.06	5.54	42.79	90.90	<0.001
MiND-B_centered (X)	−0.93	0.29	−3.24	−1.51	−0.36	0.002
AAQ-II_centered (W)	0.66	0.20	3.30	0.26	1.07	0.002
MiND-B × AAQ-II (X × W)	−0.00	0.37	−0.07	−0.08	0.07	0.942
Age (C1)	−0.04	0.16	−0.27	−0.35	0.27	0.791
Country of residence (C2)	−8.23	6.00	−1.37	−20.21	3.74	0.175
*R*^2^ = 0.37						
MSE = 160.724						
*F*(69, 5) = 8.12						
*p* < 0.001						

MiND-B: assessment of person living with MND behavioural changes; AAQ-II: assessment of carer psychological inflexibility; LLCI: lower level of 95% confident interval; ULCI: upper level of 95% confidence interval.

In practical terms, these results suggest that levels of carer psychological inflexibility independently affect their AG emotions arising from MND symptoms (i.e. higher or lower levels of carer psychological inflexibility ease or exacerbate carer AG but psychological inflexibility does not moderate the relationships between MND disease severity and behavioural changes with carer AG).

## Discussion

The present study explored two moderation models examining the role played by carer psychological inflexibility in explaining the relationship between MND symptomatology (i.e. motor and behavioural symptoms) and carer AG emotions. Results suggested that while MND symptoms and carer psychological inflexibility are associated with carer AG, psychological inflexibility as measured by the AAQ-II is not a significant moderator between MND symptoms and carer AG. These suggests that risk factors, such as MND symptoms, and buffering protective factors, such as psychological inflexibility, independently affect carer AG.

The association of disease severity and behavioural symptoms on carer AG is consistent with previous literature, underscoring these two factors as the most significant predictors of heightened levels of AG (Trucco et al., 2024b). Considering that there is no treatment available to revert MND symptomatology (McDermott and Shaw, 2008), it becomes imperative to explore alternative routes for alleviating the emotional distress experienced by carers in response to the progression of MND. A previous systematic review emphasised that the lack of, or insufficient information on MND progression negatively impacts carer AG (Trucco et al., 2023). Therefore, providing carers with information on how MND might evolve in terms of motor and behavioural symptoms, might be a proactive approach with positive impact in carers’ emotional experiences throughout the trajectory of MND. Additionally, offering carers strategies on how to address motor and behavioural symptoms might be beneficial. The MiNDToolkit, a psychoeducational intervention for the management of behavioural symptoms in MND could offer valuable support (Mioshi et al., 2024; Radakovic et al., 2020). Recommending respite care services to family carers for regular breaks from caregiving could also enhance self-care and alleviate emotional distress (Trucco et al., 2024c).

No existing study has investigated the relationship between psychological inflexibility and carer AG in MND. Findings from this study build upon current knowledge by illustrating the impact of carer psychological inflexibility on carer AG. While the results did not support the potential moderating role of psychological inflexibility, the present study demonstrated that psychological inflexibility still directly affects carer AG. A previous study demonstrated that carers may employ emotional avoidance (i.e. one form of psychological inflexibility) as a means of adjusting for lifestyle changes while refraining from disrupting feelings of AG (Trucco et al., 2023). This would suggest that emotional avoidance may act as an effective coping mechanism carers use to navigate their circumstances in the short term. However, existing research has reported that avoiding or supressing feelings and emotions, while an adaptive response to loss during acute grief responses, may, if persistent, prolong the grieving period and contribute to complicated grief (Baker et al., 2016; Eisma and Stroebe, 2021) and PGD in other carer populations (Nanni et al., 2014; Thomas et al., 2014).

The potential adverse long-term impact of emotional avoidance concerning AG emotions emphasise the importance of a comprehensive approach to undermine psychological inflexibility. Psychological interventions such as ACT can enhance psychological flexibility, which is the opposite of psychological inflexibility. ACT aims to improve one’s psychological flexibility through three sets of skills: stepping back from restricting thoughts and approaching or allowing painful emotions; focusing on the present, connecting with what is happening in the moment; and clarifying and acting on what is most important to do and building larger patterns of effective values-based actions (Hayes et al., 2013).

A recent randomised controlled trial of ACT for people living with MND demonstrated that ACT plus usual care is effective, in particular for improving psychological quality of life in patients (Gould et al., 2024). In addition, a recent systematic review (Han et al., 2021) that explored the effectiveness of ACT on mental health outcomes in family carers demonstrated that ACT is effective in reducing anxiety and depressive symptoms among the carer population, which are two psychological outcomes contributing negatively to MND carers’ AG (Trucco et al., 2023). Offering psychotherapy to MND carers is not routinely part of standard care within MND serviced in the UK despite the National Institute for Health and Care Excellence guidelines recommending support for this population during the progression of the disease (National Institute for Health and Care Excellence, 2016). Considering ACT as a potential intervention for MND carers for better emotional coping is promising.

It is essential to consider the potential avoidance of grief-related emotions in this population. As noted in the Methods section, a low score on the MMCGI-SF may reflect various psychological coping mechanisms employed by carers, including positive coping strategies or, conversely, denial of grief emotions. A low score could be indicative of denial, wherein the carer may consciously or unconsciously avoid confronting the emotional challenges of caregiving. This defence mechanism may serve to maintain emotional stability or protect against the distress associated with impending loss but could also mask underlying emotional difficulties. This distinction underscores the importance of further exploring carers’ overall emotional and psychological states more holistically. Incorporating qualitative assessments or supplementary measures could provide a more comprehensive understanding of AG, particularly as AG is a risk factor for developing PGD as mentioned previously.

While these findings are important in light of the need of providing emotional support to carers during the trajectory of MND, certain limitations should be acknowledged. Firstly, the seven-item AAQ-II was used to assess carer psychological inflexibility. Although this measure has been widely used in research as a generic measure of psychological inflexibility, questionable internal consistency has been raised and concerns about measuring global distress rather than psychological inflexibility have been reported due to its simplicity (Wolgast, 2014). Future studies should consider the inclusion of a more population-specific measure of psychological inflexibility, such as the Experiential Avoidance in Caregiving Questionnaire-EACQ (Losada et al., 2014), or a recently developed more comprehensive measure of psychological inflexibility, which covers the broader aspects of psychological inflexibility, such as the 23-item Comprehensive assessment of Acceptance and Commitment Therapy processes-CompACT (Francis et al., 2016). Secondly, the ALSFRS-R was developed primarily with input from healthcare professionals. As a result, it may not fully capture and reflect the perspectives of carers when assessing functional abilities as their emotional wellbeing could influence their perceptions and experiences. Additionally, most carers from this study presented average levels of psychological inflexibility. Future studies involving participants with diverse levels of psychological inflexibility and from different cultural backgrounds should be conducted to enhance the generalisability of the findings. Moreover, it is important to explore additional moderating factors between MND symptomatology and carer AG. For instance, emotional exhaustion, characterised by feelings of overload and emotional depletion when confronted the demands of caregiving and the needs of the care recipient, presents itself as a potential moderator factor (Gérain and Zech, 2019). It is also important to explore how other psychological variables, such as depression and anxiety influence AG and its relationship with MND symptoms. Given that AG often encompasses elements of anxiety and depression (Rando, 2000; Simon, 2008), and previous research has reported high level of these conditions among MND carers (Gauthier et al., 2007; Vignola et al., 2008), their role warrants further investigation. We also acknowledge that elevated levels of anxiety and depression may have influenced participant’s responses on the self-rating measures, representing a potential limitation of this study. Additionally, conducting further studies on how cognitive symptoms in the person living with MND may affect carer AG and longitudinal studies to account for disability progression and how this might affect AG should be considered. It is important to acknowledge that while including country of residence as a covariate to control for potential confounding effects of country of residence on carer anticipatory grief, the imbalance in sample sizes could affect the interpretation and generalisation of results. Future research with a more balanced distribution of participants from different countries could offer deeper insights into the relationships between variables. Moreover, studies that include participants from diverse ethnicities, education levels and other relationships with the care recipient, as well as exploring AG between genders are needed to improve the representativeness and generalisation of the data.

Notwithstanding the limitations outlined above, the findings of this study are remarkably novel. To our knowledge, there is a lack of research exploring the moderating effect of psychological inflexibility on this population, thereby contributing significantly to the existing body of literature in this field.

### Conclusion

This study suggested that carer psychological inflexibility does not moderate the relationship between MND disease severity and behavioural symptoms, and carer AG. Nevertheless, it is noteworthy that all three factors – disease severity, behavioural changes and carer psychological inflexibility – are individually associated with AG. These findings suggest ACT could be a potential intervention for addressing and enhancing psychological flexibility. In addition, further studies are needed to explore modifiable factors that could contribute to ease carers’ feelings of losses and changes.

## References

[bibr1-13591053251328490] Abrahams S (2023) Neuropsychological impairment in amyotrophic lateral sclerosis-frontotemporal spectrum disorder. Nature Reviews Neurology 19(11): 655–667. 37828358 10.1038/s41582-023-00878-z

[bibr2-13591053251328490] Abrahams S Leigh PN Harvey A , et al. (2000) Verbal fluency and executive dysfunction in amyotrophic lateral sclerosis (ALS). Neuropsychologia 38(6): 734–747. 10689049 10.1016/s0028-3932(99)00146-3

[bibr3-13591053251328490] Aoun SM Breen LJ Howting DA , et al. (2015) Who needs bereavement support? A population based survey of bereavement risk and support need. PLoS One 10(3): e0121101. 10.1371/journal.pone.0121101 PMC4374848 25811912

[bibr4-13591053251328490] Baker AW Keshaviah A Horenstein A , et al. (2016) The role of avoidance in complicated grief: A detailed examination of the Grief-Related Avoidance Questionnaire (GRAQ) in a large sample of individuals with complicated grief. Journal of Loss and Trauma 21(6): 533–547. 28649184 10.1080/15325024.2016.1157412 PMC5482544

[bibr5-13591053251328490] Bond FW Hayes SC Baer RA , et al. (2011) Preliminary psychometric properties of the Acceptance and Action Questionnaire-II: A revised measure of psychological inflexibility and experiential avoidance. Behaviour Therapy 42(4): 676–688. 10.1016/j.beth.2011.03.007 22035996

[bibr6-13591053251328490] Cedarbaum JM Stambler N Malta E. , et al; BDNF ALS Study Group (Phase III) (1999) The ALSFRS-R: A revised ALS functional rating scale that incorporates assessments of respiratory function. Journal of the Neurological Sciences 169(1–2): 13–21. 10540002 10.1016/s0022-510x(99)00210-5

[bibr7-13591053251328490] Coelho A Barbosa A (2017) Family anticipatory grief: An integrative literature review. American Journal of Hospice and Palliative Care 34(8): 774–785. 27151972 10.1177/1049909116647960

[bibr8-13591053251328490] Coelho A de Brito M Barbosa A (2018) Caregiver anticipatory grief: Phenomenology, assessment and clinical interventions. Current Opinion in Supportive and Palliative Care 12(1): 52–57. 29206700 10.1097/SPC.0000000000000321

[bibr9-13591053251328490] Eisma MC Stroebe MS (2021) Emotion regulatory strategies in complicated grief: A systematic review. Behaviour Therapy 52(1): 234–249. 10.1016/j.beth.2020.04.004 33483120

[bibr10-13591053251328490] Francis AW Dawson DL Golijani-Moghaddam N (2016) The development and validation of the Comprehensive assessment of Acceptance and Commitment Therapy processes (CompACT). Journal of Contextual Behavioral Science 5(3): 134–145.

[bibr11-13591053251328490] Gauthier A Vignola A Calvo A , et al. (2007) A longitudinal study on quality of life and depression in ALS patient-caregiver couples. Neurology 68: 923–926. 17372127 10.1212/01.wnl.0000257093.53430.a8

[bibr12-13591053251328490] Gérain P Zech E (2019) Informal caregiver burnout? Development of a theoretical framework to understand the impact of caregiving. Frontiers in Psychology 10: 1748. 31428015 10.3389/fpsyg.2019.01748 PMC6689954

[bibr13-13591053251328490] Gould RL McDermott CJ Thompson BJ , et al. (2024) Acceptance and Commitment Therapy plus usual care for improving quality of life in people with motor neuron disease (COMMEND): A multicentre, parallel, randomised controlled trial in the UK. The Lancet 403(10442): 2381–2394. 10.1016/S0140-6736(24)00533-6 38735299

[bibr14-13591053251328490] Han A Yuen HK Jenkins J (2021) Acceptance and commitment therapy for family caregivers: A systematic review and meta-analysis. Journal of Health Psychology 26(1): 82–102. 32659142 10.1177/1359105320941217

[bibr15-13591053251328490] Hardiman O Al-Chalabi A Chio A , et al. (2017) Amyotrophic lateral sclerosis. Nature Reviews Disease Primers 3: 17071. 10.1038/nrdp.2017.71 28980624

[bibr16-13591053251328490] Hayes A (2013) Introduction to Mediation, Moderation and Conditional Process Analysis: A Regression-Based Approach. London & New York: Guilford Press.

[bibr17-13591053251328490] Hayes SC Levin ME Plumb-Vilardaga J , et al. (2013) Acceptance and commitment therapy and contextual behavioral science: Examining the progress of a distinctive model of behavioral and cognitive therapy. Behaviour Therapy 44(2): 180–198. 10.1016/j.beth.2009.08.002 PMC3635495 23611068

[bibr18-13591053251328490] Hayes SC Luoma JB Bond FW , et al. (2006) Acceptance and commitment therapy: Model, processes and outcomes. Behavior Research Therapy 44(1): 1–25. 10.1016/j.brat.2005.06.006 16300724

[bibr19-13591053251328490] Jones K Methley A Boyle G , et al. (2022) A systematic review of the effectiveness of acceptance and commitment therapy for managing grief experienced by bereaved spouses or partners of adults who had received palliative care. Illness, Crisis and Loss 30(4): 596–613.

[bibr20-13591053251328490] Karekla M Panayiotou G (2011) Coping and experiential avoidance: Unique or overlapping constructs? Journal of Behavior Therapy and Experimental Psychiatry 42(2): 163–170. 21315877 10.1016/j.jbtep.2010.10.002

[bibr21-13591053251328490] Kiernan MC Vucic S Cheah BC , et al. (2011) Amyotrophic lateral sclerosis. The Lancet 377(9769): 942–955. 10.1016/S0140-6736(10)61156-7 21296405

[bibr22-13591053251328490] Kustanti CY Chu H Kang XL , et al. (2022) Anticipatory grief prevalence among caregivers of persons with a life-threatening illness: A meta-analysis. BMJ Supportive and Palliative Care 13(3): 1074–1083. 10.1136/bmjspcare-2021-003338 35149523

[bibr23-13591053251328490] Losada A Márquez-González M Romero-Moreno R , et al. (2014) Development and validation of the Experiential Avoidance in Caregiving Questionnaire (EACQ). Aging and Mental Health 18(7): 897–904. 24678984 10.1080/13607863.2014.896868

[bibr24-13591053251328490] McDermott CJ Shaw PJ (2008) Diagnosis and management of motor neurone disease. British Medical Journal 336(7645): 658–662. 18356234 10.1136/bmj.39493.511759.BE PMC2270983

[bibr25-13591053251328490] Marwit SJ Meuser TM (2005) Development of a short form inventory to assess grief in caregivers of dementia patients. Death Studies 29(3): 191–205. 15816111 10.1080/07481180590916335

[bibr26-13591053251328490] Mehdipour A Teshler L Dal Bello-Haas V , et al. (2023) Assessing the measurement properties of the self-administered Amyotrophic Lateral Sclerosis Functional Rating Scale-Revised (ALSFRS-R): A Rasch analysis. Physical Therapy 103(11): pzad109. 10.1093/ptj/pzad109 37581600

[bibr27-13591053251328490] Meuser TM Marwit SJ (2001) A comprehensive, stage-sensitive model of grief in dementia caregiving. The Gerontologist 41(5): 658–670. 11574711 10.1093/geront/41.5.658

[bibr28-13591053251328490] Meyer T Spittel S Grehl T , et al. (2023) Remote digital assessment of amyotrophic lateral sclerosis functional rating scale - A multicenter observational study. Amyotrophic Lateral Sclerosis and Frontotemporal Degeneration 24(3–4): 175–184. 35912984 10.1080/21678421.2022.2104649

[bibr29-13591053251328490] Miano B Stoddard GJ Davis S , et al. (2004) Inter-evaluator reliability of the ALS functional rating scale. Amyotrophic Lateral Sclerosis and Other Motor Neuron Disorders 5(4): 235–239. 15799553 10.1080/14660820410021302

[bibr30-13591053251328490] Mioshi E Grant K Flanagan E , et al. (2024) An online intervention for carers to manage behavioural symptoms in motor neurone disease (MiNDToolkit): A randomised parallel multi-centre feasibility trial. Amyotrophic Lateral Sclerosis and Frontotemporal Degeneration 25(5–6): 506–516. 38745522 10.1080/21678421.2024.2350658 PMC11286211

[bibr31-13591053251328490] Mioshi E Hsieh S Caga J , et al. (2014) A novel tool to detect behavioural symptoms in ALS. Amyotrophic Lateral Sclerosis and Frontotemporal Degeneration 15(3–4): 298–304. 24863641 10.3109/21678421.2014.896927

[bibr32-13591053251328490] Montes J Levy G Albert S , et al. (2006) Development and evaluation of a self-administered version of the ALSFRS-R. Neurology 67(7): 1294–1296. 17030772 10.1212/01.wnl.0000238505.22066.fc

[bibr33-13591053251328490] Nanni MG Biancosino B Grassi L (2014) Pre-loss symptoms related to risk of complicated grief in caregivers of terminally ill cancer patients. Journal of Affective Disorders 160: 87–91. 24445130 10.1016/j.jad.2013.12.023

[bibr34-13591053251328490] National Institute for Health and Care Excellence (2016) Motor neurone disease: Assessment and management (NG42). Available at: www.nice.org.uk/Guidance/NG42 (accessed 5 June 2024). 26962594

[bibr35-13591053251328490] Nielsen MK Neergaard MA Jensen AB , et al. (2016) Do we need to change our understanding of anticipatory grief in caregivers? A systematic review of caregiver studies during end-of-life caregiving and bereavement. Clinical Psychology Review 44: 75–93. 26796738 10.1016/j.cpr.2016.01.002

[bibr36-13591053251328490] Ottenbreit ND Dobson KS (2004) Avoidance and depression: The construction of the cognitive-behavioral avoidance scale. Behaviour Research and Therapy 42(3): 293–313. 14975771 10.1016/S0005-7967(03)00140-2

[bibr37-13591053251328490] Pinto C Geraghty AWA Yardley L , et al. (2021) Emotional distress and well-being among people with motor neurone disease (MND) and their family caregivers: A qualitative interview study. British Medical Journal Open 11(8): e044724. 10.1136/bmjopen-2020-044724 PMC8372816 34404695

[bibr38-13591053251328490] Radakovic R Copsey H Moore C , et al. (2020) Development of the MiNDToolkit for management of cognitive and behavioral impairment in motor neuron disease. Neurodegenerative Disease Management 10(1): 15–25. 31973641 10.2217/nmt-2019-0035

[bibr39-13591053251328490] Rando TA (1986) A comprehensive analysis of anticipatory grief: Perspectives, process, promises, and problems. In: Rando TA (ed.) Loss and Anticipatory Grief. New York, NY: Lexington Books, pp.3–37.

[bibr40-13591053251328490] Rando TA (2000) Anticipatory mourning: A review and critique of the literature. In: Rando TA (ed.) Clinical Dimensions of Anticipatory Mourning: Theory and Practice in Working With the Dying, Their Loved Ones, and Their Caregivers. Champaign, IL: Research Press, pp.17–50.

[bibr41-13591053251328490] Shrestha N (2020) Detecting multicollinearity in regression analysis. American Journal of Applied Mathematics and Statistics 8: 39–42.

[bibr42-13591053251328490] Simon JL (2008) Anticipatory grief: Recognition and coping. Journal of Palliative Medicine 11(9): 1280–1281. 19021499 10.1089/jpm.2008.9824

[bibr43-13591053251328490] Statland JM Barohn RJ McVey AL , et al. (2015) Patterns of weakness, classification of motor neuron disease, and clinical diagnosis of sporadic amyotrophic lateral sclerosis. Neurologic Clinics 33(4): 735–748. 26515618 10.1016/j.ncl.2015.07.006 PMC4629510

[bibr44-13591053251328490] Strong MJ Abrahams S Goldstein LH , et al. (2017) Amyotrophic lateral sclerosis - frontotemporal spectrum disorder (ALS-FTSD): Revised diagnostic criteria. Amyotrophic Lateral Sclerosis and Frontotemporal Degeneration 18(3–4): 153–174. 28054827 10.1080/21678421.2016.1267768 PMC7409990

[bibr45-13591053251328490] Thomas K Hudson P Trauer T , et al. (2014) Risk factors for developing prolonged grief during bereavement in family carers of cancer patients in palliative care: A longitudinal study. Journal of Pain and Symptom Management 47(3): 531–541. 23969327 10.1016/j.jpainsymman.2013.05.022

[bibr46-13591053251328490] Trucco AP Backhouse T Mioshi E (2024a) Describing and assessing behavioural symptoms in amyotrophic lateral sclerosis with and without frontotemporal dementia: A scoping review. Current Opinion in Neurology 37(5): 603–610. 38946579 10.1097/WCO.0000000000001293

[bibr47-13591053251328490] Trucco AP Backhouse T Mioshi E , et al. (2023) Factors associated with grief in informal carers of people living with Motor Neuron Disease: A mixed methods systematic review. Death Studies 48(2): 103–117. 36995270 10.1080/07481187.2023.2191351 PMC11601043

[bibr48-13591053251328490] Trucco AP Khondoker M Kishita N , et al. (2024b) Factors affecting anticipatory grief of family carers currently supporting people living with Motor Neurone Disease: The impact of disease symptomatology. Amyotrophic Lateral Sclerosis and Frontotemporal Degeneration 25(7–8): 776–784. 38813983 10.1080/21678421.2024.2359559 PMC11523914

[bibr49-13591053251328490] Trucco AP Mioshi E Kishita N , et al. (2024c) Navigating an emotional journey: A qualitative study of the emotional experiences of family carers currently supporting people living with motor neurone disease. Palliative and Supportive Care 22(5): 1191–1197. 37935447 10.1017/S147895152300158X

[bibr50-13591053251328490] Vignola A Guzzo A Calvo A , et al. (2008) Anxiety undermines quality of life in ALS patients and caregivers. European Journal of Neurology 15(11): 1231–1236. 18803649 10.1111/j.1468-1331.2008.02303.x

[bibr51-13591053251328490] Wolgast M (2014) What does the Acceptance and Action Questionnaire (AAQ-II) really measure? Behavior Therapy 45(6): 831–839. 25311291 10.1016/j.beth.2014.07.002

